# The prognostic value of circulating tumor DNA in malignant melanoma patients treated with immune checkpoint inhibitors: a systematic review and meta-analysis

**DOI:** 10.3389/fimmu.2024.1520441

**Published:** 2025-01-17

**Authors:** Lei Liu, Shufu Hou, Aiping Zhu, Bing Yan, Linchuan Li, Dandan Song

**Affiliations:** ^1^ Department of Neurology, Shandong Provincial Third Hospital, Cheeloo College of Medicine, Shandong University, Jinan, China; ^2^ Department of Gastrointestinal Surgery, Central Hospital Affiliated to Shandong First Medical University, Jinan, China; ^3^ Department of Neurology, Shandong Second Provincial General Hospital, Jinan, China; ^4^ Department of General Surgery, The First Affiliated Hospital of Shandong First Medical University, Jinan, China

**Keywords:** circulating tumor DNA, immune checkpoint inhibitors, malignant melanoma, overall survival, progression-free survival

## Abstract

**Background:**

Circulating tumor DNA (ctDNA) is an emerging biomarker in malignant melanoma(MM), and high levels of ctDNA may reflect a higher tumor load. However, its prognostic value for MM receiving immune checkpoint inhibitors(ICI) remains controversial. This meta-analysis aimed to elucidate the prognostic significance of ctDNA in this patient population.

**Methods:**

We conducted a comprehensive search of the PubMed, Cochrane Library, CNKI, and EMBASE databases, including studies published up to August 15, 2024, to investigate the prognostic impact of ctDNA in MM patients treated with ICI. Using a fixed-effects model, we systematically evaluated the association between ctDNA levels and key survival outcomes, including overall survival (OS) and progression-free survival (PFS). Additionally, funnel plots, Begg’s test, and Egger’s test were employed to assess potential publication bias.

**Results:**

Twelve studies from eleven articles, involving a total of 1063 eligible MM patients receiving ICI therapy, were included. The results indicated that patients with detectable ctDNA before initiating ICI therapy had significantly poorer OS (HR = 3.19, 95% CI = 2.22–4.58, P < 0.001) and PFS (HR = 2.08, 95% CI = 1.61–2.69, P < 0.001). Furthermore, the detectability of ctDNA during treatment was also significantly associated with worse OS (HR = 4.57, 95% CI = 3.03–6.91, P < 0.001) and PFS (HR = 3.79, 95% CI = 2.13–6.75, P < 0.001).

**Conclusions:**

This meta-analysis indicates that in MM patients receiving ICI therapy, detectable and high levels of ctDNA are significantly associated with poorer OS and PFS. Therefore, ctDNA can serve as a diagnostic and stratification tool prior to treatment, as well as an effective indicator for monitoring treatment response and disease progression.

**Systematic Review Registration:**

www.inplasy.com, identifier INPLASY2024110018.

## 1 Introduction

Malignant melanoma (MM) is an aggressive and fatal tumor caused by the abnormal proliferation of melanocytes (1). In its early stages, MM is typically confined to the skin and presents with nonspecific symptoms, often delaying diagnosis. Advanced MM, however, progresses rapidly, metastasizes early, and has a poor prognosis (2–4). The global incidence of MM has been steadily increasing in recent years (5). Surgery remains the primary treatment for early-stage localized MM, but traditional chemotherapy and radiotherapy have shown limited efficacy in advanced or metastatic cases (6, 7). For instance, dacarbazine, once a standard chemotherapy for advanced MM, has demonstrated low response rates and minimal survival benefits (8). In the 21st century, immunotherapy has revolutionized MM treatment due to its unique susceptibility to immune modulation. ICI have significantly improved OS in patients with advanced MM (9–11). However, 40–60% of patients fail to respond to immunotherapy (12).

Unlike other solid tumors, MM lacks highly specific serum biomarkers, complicating effective monitoring. Traditional tissue biopsies are unsuitable for continuous surveillance. Liquid biopsy, a minimally invasive and highly sensitive approach, has recently gained traction in MM (13, 14). Identifying serum biomarkers with high sensitivity and specificity is critical for improving early diagnosis, real-time disease monitoring, and personalized treatment strategies. PD-L1 expression on tumor cells is currently the most accepted predictive biomarker and is associated with a higher likelihood of treatment response (15). However, PD-L1 expression exhibits significant heterogeneity, and even PD-L1-negative patients can achieve response rates of up to 41.3% (16). This underscores the need for more reliable biomarkers to predict immunotherapy outcomes and minimize unnecessary immune-related adverse effects.

Circulating tumor DNA (ctDNA), consisting of tumor-derived genetic material such as mutations, gene rearrangements, and copy number variations, has emerged as a promising noninvasive biomarker through liquid biopsy (17, 18). Studies have demonstrated the prognostic value of ctDNA in other cancers. For instance, ctDNA detection correlates with poor OS and PFS in metastatic breast cancer (19), poor recurrence-free survival and OS in early-stage breast cancer (20), and promising efficacy and prognosis assessments in locally advanced rectal cancer treated with neoadjuvant chemoradiotherapy (21–23). However, the role of ctDNA in predicting immunotherapy response and prognosis in MM patients treated with ICI remains unclear. To address this, we conducted a systematic meta-analysis of published literature to evaluate the prognostic value of ctDNA in MM patients undergoing ICI therapy, aiming to inform future clinical applications.

## 2 Materials and methods

### 2.1 Search strategy

This systematic review and meta-analysis were conducted in accordance with the guidelines outlined in the Preferred Reporting Items for Systematic Reviews and Meta-Analyses (PRISMA) (24). Two independent researchers systematically searched PubMed, Embase, CNKI, and the Cochrane Library to identify studies related to the prognostic significance of ctDNA in MM patients receiving ICI therapy. The search encompassed relevant studies from the inception of these databases until August 15, 2024. We utilized the following keywords to investigate the predictive significance of ctDNA in MM patients treated with ICI: “Melanoma” or “Melanomas” or “Malignant Melanomas” or “Malignant Melanoma” and “ctDNA” or “circulating tumor DNA” and “PD-L1 inhibitors” or “immune checkpoint inhibitors” or “programmed cell death ligand-1 inhibitors” or “immunotherapy”. In addition to employing free-text terms and Medical Subject Headings (MeSH) for searching within titles and abstracts, we also screened the references of selected articles to ensure comprehensive retrieval.

### 2.2 Inclusion and exclusion criteria

#### 2.2.1 Inclusion criteria

(1) Patients with unresectable, previously untreated stage III or IV melanoma, confirmed through gold standard pathological diagnosis, who are receiving systemic treatment with immune checkpoint inhibitors; (2) Clinical studies related to the prognostic value of circulating tumor DNA; (3) Studies that provide direct or indirect outcomes related to OS and PFS for MM patients, including hazard ratios (HR) and 95% confidence intervals (CI).

#### 2.2.2 Exclusion criteria

(1) Patients with a diagnosis of melanoma alongside uveal melanoma or other primary cancers; (2) Studies focused solely on cell-free DNA data; (3) Case reports, conference abstracts, animal studies, or reviews; (4) Studies lacking sufficient and valid data to estimate HR and 95% CI; (5) Duplicate publications of data.

### 2.3 Data extraction and quality assessment

Two independent researchers extracted data from the eligible studies, with any discrepancies resolved through discussion or consultation with a third researcher. The extracted data included the first author’s name, publication year, study location, study design, sample size, mean or median patient age, cancer stage, treatment methods, detection techniques, timing of sample collection, target genes, median follow-up period (in months), survival analysis (including hazard ratios and 95% confidence intervals for OS and PFS). Study quality was assessed using the Newcastle-Ottawa Scale (NOS), which evaluates three key domains: selection (0–4 points), comparability (0–2 points), and outcome assessment (0–3 points). Each researcher independently scored the eight questions across these domains, with a total possible score ranging from 0 to 9. Studies scoring more than 6 points were classified as high quality (25).

### 2.4 Statistical methods

The statistical analysis for this study was performed using Stata SE (version 16.0; StataCorp, College Station, Texas, USA). Hazard ratios (HR) with 95% confidence intervals (CI) were used to evaluate the potential association between ctDNA and OS as well as PFS. We provide two types of hazard ratios (HR) derived from pooling HR estimates under the following conditions: (a) ctDNA measured at baseline, prior to surgery or any other form of treatment; and (b) ctDNA measured either once or multiple times after the initiation of ICI therapy. This distinction allows for a clear analysis of the timing of ctDNA measurements in relation to treatment, providing insights into their predictive value at different stages of patient management. Heterogeneity among the studies was assessed using Cochran’s Q-test and I² statistics. Based on these results, an appropriate effect model was selected. If I² > 50% or the p-value from the Q-test was < 0.10, indicating significant heterogeneity, a random-effects model was applied. Otherwise, a fixed-effects model was used. Publication bias was assessed by examining the symmetry of the funnel plot, alongside statistical methods such as Egger’s linear regression and Begg’s test, with a p-value < 0.05 suggesting publication bias. Sensitivity analyses were also conducted to evaluate the impact of individual studies on OS and PFS.

## 3 Results

### 3.1 Study selection and characteristics

**Figure 1:**
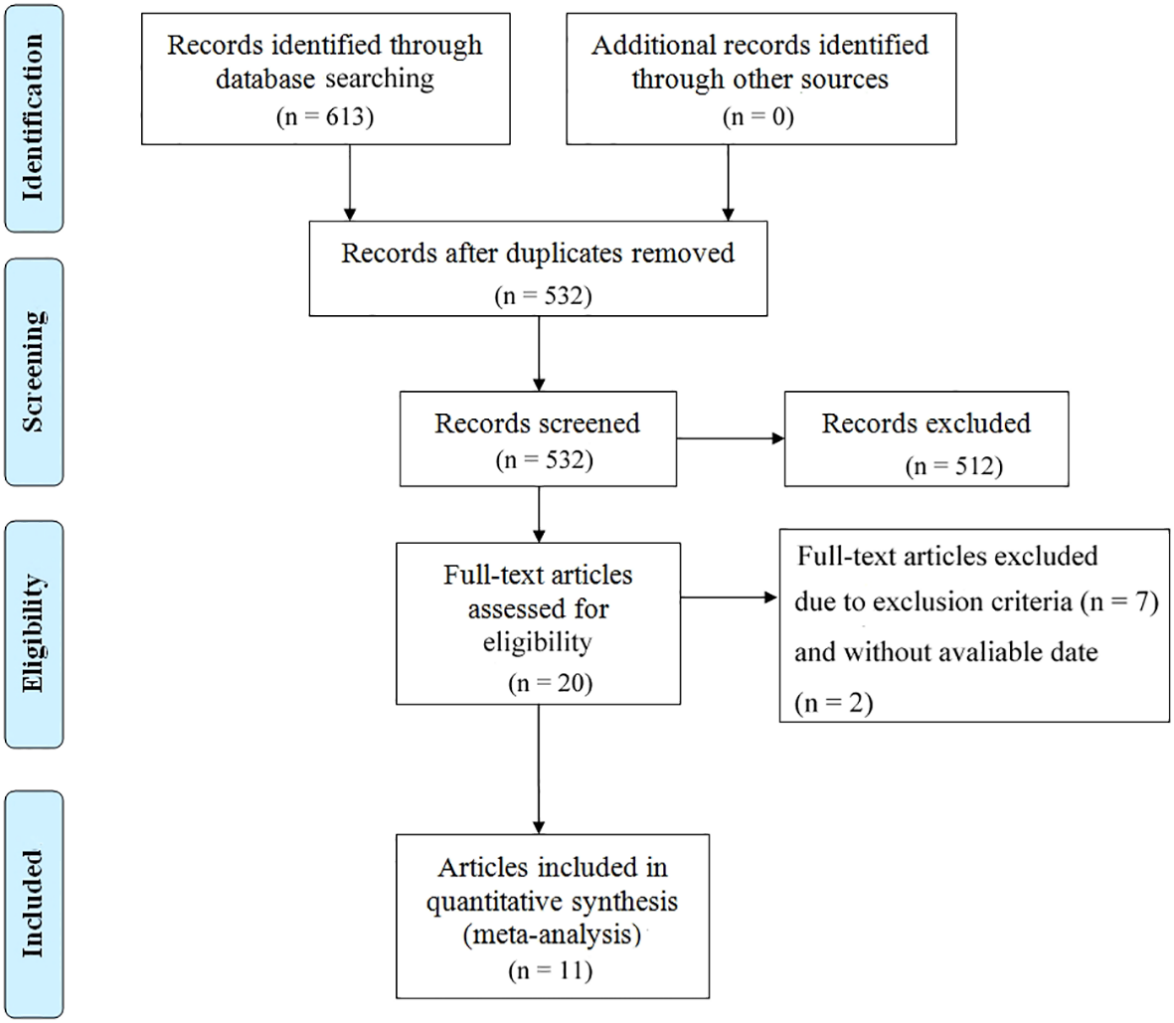
Prisma flowchart illustrating the process of literature selection.

The study selection process is illustrated in **Figure 1**. A total of 613 articles were initially retrieved, including 133 from PubMed, 499 from Embase, and 25 from The Cochrane Library. After removing duplicates, 532 articles remained. Following a detailed screening of titles and abstracts based on predefined inclusion and exclusion criteria, 492 articles were excluded. Additionally, two articles were excluded due to the unavailability of full text. Ultimately, 11 articles representing 12 observational cohort studies were included (26–36). The characteristics of the included studies are summarized in **Table 1**. All studies were published between 2018 and 2023, with four studies conducted in Australia (represented by three articles), two in France, two in Belgium, two in the United States, one in Denmark, and one in Germany. Sample sizes ranged from 16 to 267 patients, with a total of 1,063 cases included. Five studies reported OS prior to initiating ICI treatment, and five reported OS during ICI treatment. Additionally, six studies reported PFS prior to treatment, while four studies reported PFS during treatment. According to the Newcastle-Ottawa Quality Assessment Scale (NOS), the included studies scored between 7 and 8, indicating a relatively high level of data quality. Detailed NOS scores for all included articles are provided in **Table 2**.

**Table 1:** Baseline characteristics of included studies.

Study, year	Country	Sample size	Median age	Gender (M/F)	Stage	ICIs used	Detection methods	Time of sample collection	Target genes	Median follow-up (months)	Survival outcome	NOS
Herbreteau 2018 (35)	France	53	64 (27–90)	29/24	III-IV	Nivolumab ipilimumab	dPCR	Pretreatment	BRAF,NRAS	10.2-25.2	OS,PFS	7
Forschner 2019 (31)	Germany	35	55	19/16	IV	Nivolumab ipilimumab	ddPCR	Posttreatment	BRAF	7.1	OS	7
Seremet 2019 (32)	Belgium	85	57 (27–82)	37/48	III-IV	pembrolizumab	ddPCR	Pretreatment Posttreatment	BRAF,NRAS	21	OS,PFS	8
Lee 2020 (34)	Australia	72	NR	49/23	NR	nivolumab,ipilimumab pembrolizumab	NR	Pretreatment Posttreatment	BRAF, NRAS, c-KIT	35.6 (3.7-50.8)	OS	7
Pedersen 2020 (33)	Denmark	16	57 (47-75)	11/5	III-IV	nivolumab,ipilimumab pembrolizumab	ddPCR	Posttreatment	BRAF,TERT	26 (6.3-35.6)	PFS	7
Marsavela 2020 1 (29)	Australia	59	NR	45/14	IV	nivolumab,ipilimumab pembrolizumab	ddPCR	Pretreatment	BRAF	23.75 (4-64.25)	PFS	7
Marsavela 2020 2 (29)	Australia	128	NR	83/45	III-IV	nivolumab,ipilimumab pembrolizumab	ddPCR	Pretreatment	BRAF	NR	PFS	6
Awada 2021 (26)	Belgium	183	60 (24–93)	88/95	III-IV	pembrolizumab	ddPCR	Pretreatment	BRAFV600 or NRASQ61/G12/G13	52.7	OS,PFS	7
Herbreteau 2021 (28)	France	102	63	45/57	III-IV	Nivolumab ipilimumab	dPCR	Pretreatment	BRAF or NRAS	10.8 (0.7-42)	OS,PFS	7
Eroglu 2023 (27)	USA	29	64 (39–89)	20/9	III-IV	Nivolumab ipilimumab	mPCR	Posttreatment	NR	14.2 (0.2–20.8)	PFS	7
Tawbi 2022 (36)	USA	267	56	NR	III-IV	spartalizumab	NR	Posttreatment	BRAF	27.2	OS,PFS	7
Warburton 2023 (30)	Australia	34	53 (20–86)	21/13	IV	Nivolumab ipilimumab	ddPCR	Posttreatment	BRAF	NR	OS,PFS	6

NR, not report; ICIs, immune checkpoint inhibitors; OS, overall survival; PFS, progression-free survival; ddPCR, droplet digital polymerase chain reactionmultivariate; mPCR, multiplex polymerase chain reaction; NOS, Newcastle-Ottawa Scale.

**Table 2:** Newcastle-Ottawa Scale (NOS) for quality assessment.

Studies	Selection				Comparability	Outcome			Scores
	A	B	C	D	E	F	G	H	
Herbreteau 2018 (35)	★	★	★	★	★	★	★	–	7
Forschner 2019 (31)	★	★	★	★	★	★	★	–	7
Seremet 2019 (32)	★	★	★	★	★★	★	★	–	8
Lee 2020 (34)	★	★	★	★	★	★	★	–	7
Pedersen 2020 (33)	★	★	★	★	★	★	★	–	7
Marsavela 2020 1 (29)	★	★	★	★	★	★	★	–	7
Marsavela 2020 2 (29)	★	★	★	★	★	★	–	–	6
Awada 2021 (26)	★	★	★	★	★	★	★	–	7
Herbreteau 2021 (28)	★	★	★	★	★	★	★	–	7
Eroglu 2023 (27)	★	★	★	★	★	★	★	–	7
Tawbi 2022 (36)	★	★	★	★	★	★	★	–	7
Warburton 2023 (30)	★	★	★	★	★	★	–	–	6

A study may receive a maximum of one star for each numbered item in the Selection and Outcome categories. A maximum of two stars may be given for Comparability, as directed by the NOS. ★ It stands for one point; ★★ It stands for two points.

### 3.2 Association of ctDNA with OS and PFS

**Figure 2:**
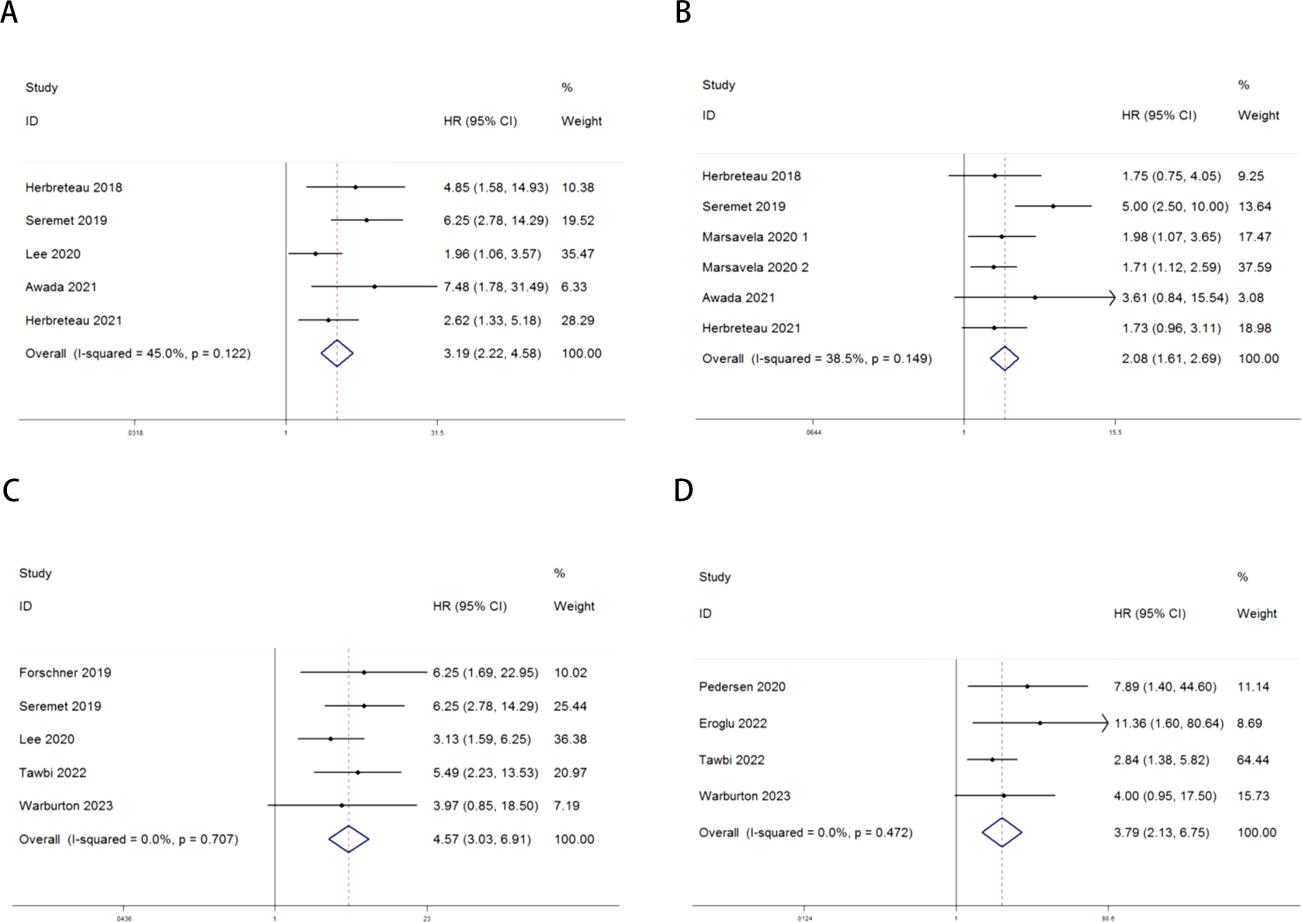
Forest plots for the association between ctDNA levels and OS and PFS in MM patients prior to and during ICI therapy (Pre-treatment: OS: **A**; PFS: **B**; Post-treatment: OS: **C**; PFS: **D**).

The relationship between ctDNA and overall survival (OS) as well as progression-free survival (PFS) in MM patients receiving immune checkpoint inhibitors (ICIs) is as follows: Heterogeneity testing indicated no significant heterogeneity (Pre-treatment: OS: P = 0.122 > 0.1, I² = 45% < 50%; PFS: P = 0.149 > 0.1, I² = 38.5% < 50%; Post-treatment: OS: P = 0.707 > 0.1, I² = 0.0% < 50%; PFS: P = 0.472 > 0.1, I² = 0.0% < 50%), suggesting that the fixed-effects model was appropriate for this meta-analysis. Independent risk estimates from five studies, along with six estimates from another five studies, demonstrated that MM patients with detectable baseline ctDNA or ctDNA levels above a specific threshold prior to ICI treatment had significantly worse OS (**Figure 2A**) and PFS (**Figure 2B**) compared to those with undetectable ctDNA. The pooled hazard ratios (HRs) and their 95% confidence intervals (CIs) were as follows: OS: HR = 3.19, 95% CI = 2.22–4.58, P < 0.001; PFS: HR = 2.08, 95% CI = 1.61–2.69, P < 0.001.Similarly, independent risk estimates from an additional five and four studies demonstrated that higher ctDNA levels after ICI treatment were significantly associated with poorer OS (**Figure 2C**) and PFS (**Figure 2D**) in MM patients. The pooled HRs and their 95% CIs were as follows: OS: HR = 4.57, 95% CI = 3.03–6.91, P < 0.001; PFS: HR = 3.79, 95% CI = 2.13–6.75, P < 0.001. Heterogeneity testing for these analyses also indicated no significant heterogeneity.

### 3.3 Publication bias

**Figure 3:**
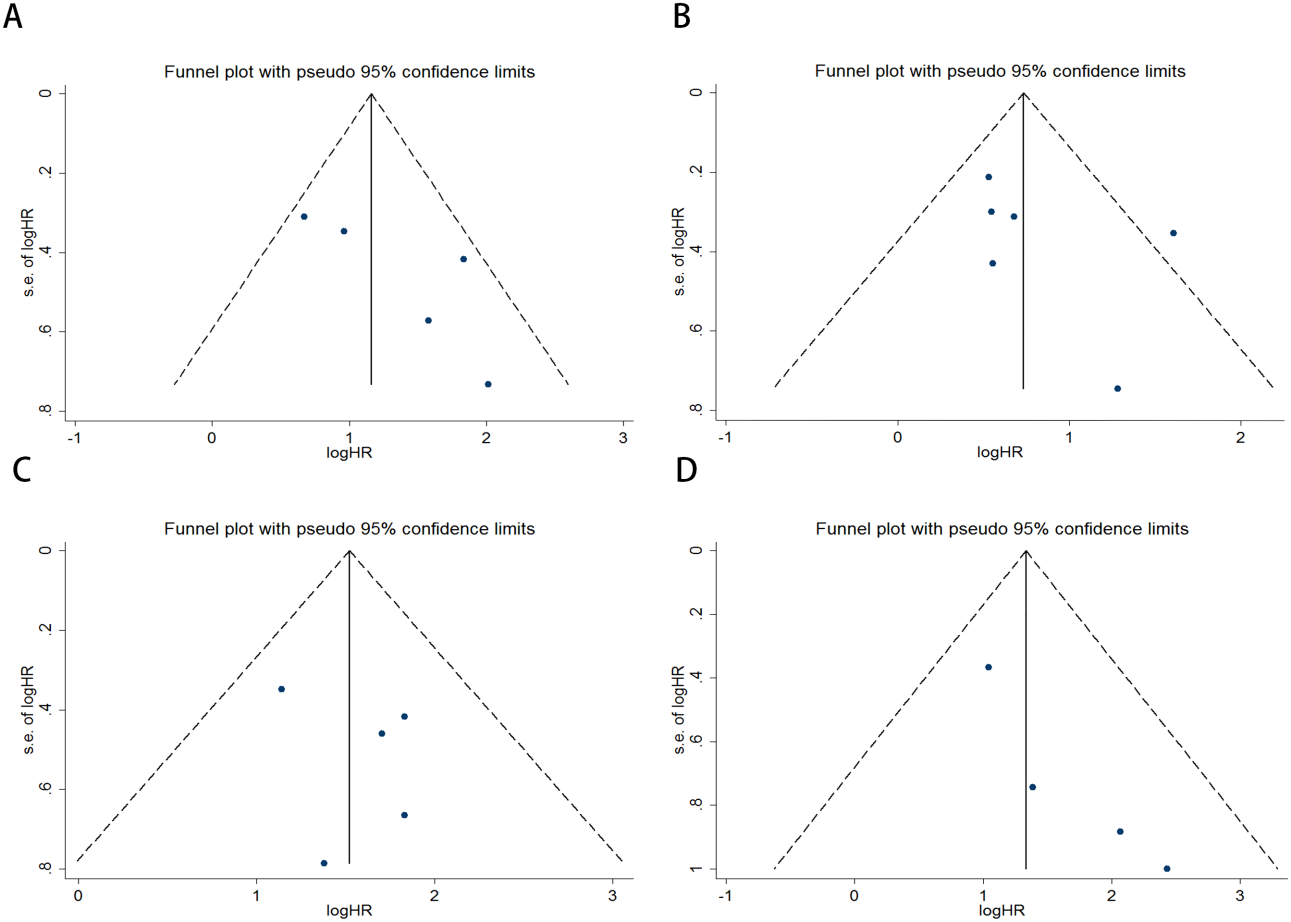
Funnel plots used to assess the presence of publication bias in MM patients receiving ICI therapy, including **(A)** OS and **(B)** PFS prior to treatment, and **(C)** OS and **(D)** PFS following treatment.

**Figure 4:**
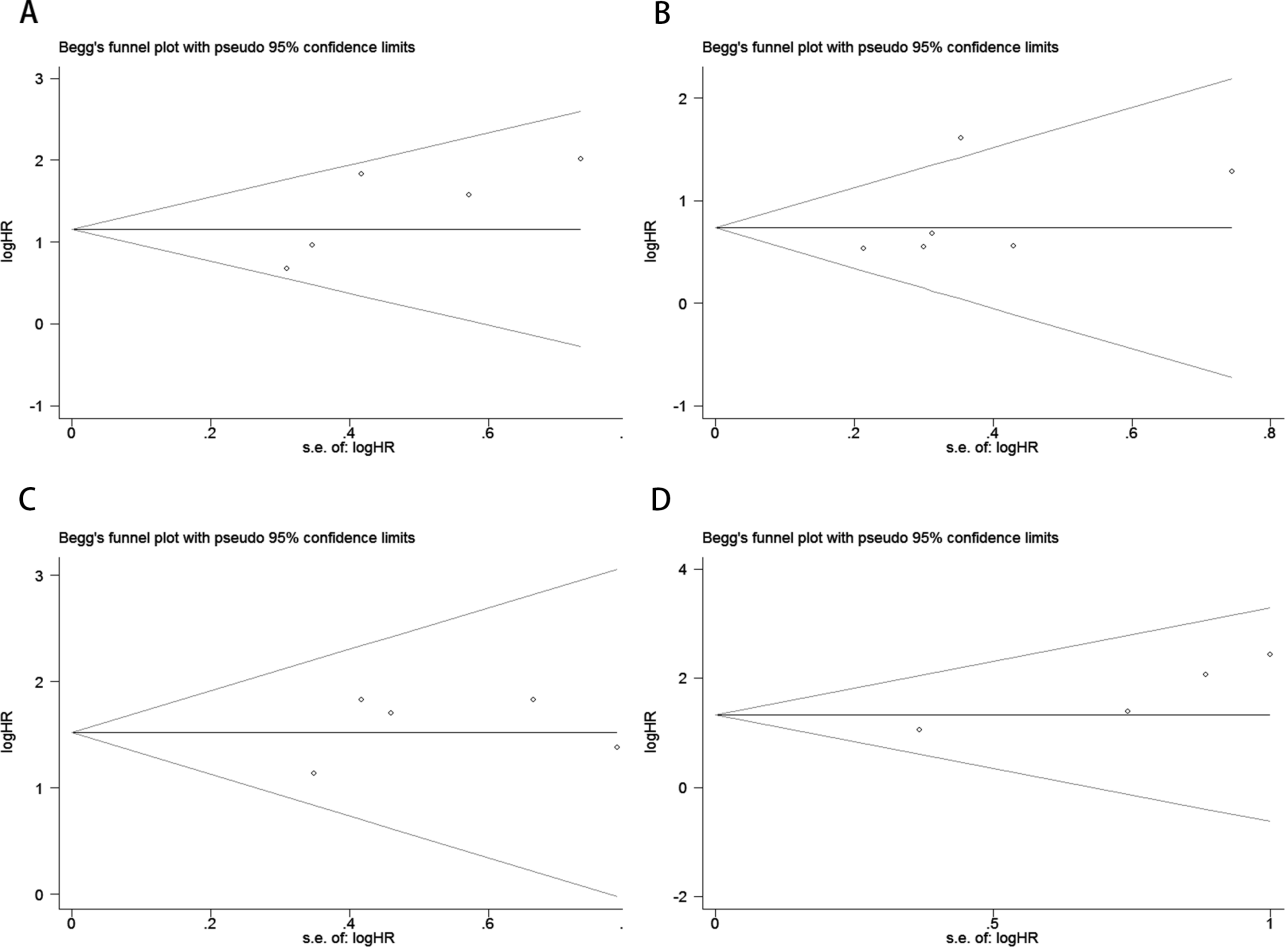
Publication bias test. **(A)** Begg tests for OS before ICI treatment, p = 0.221; **(B)** Begg tests for PFS before ICI treatment, p = 0.133; **(C)** Begg tests for OS after receiving ICI therapy.p = 1.000; **(D)** Begg tests for PFS after receiving ICI therapy. p = 0.089.

**Figure 5:**
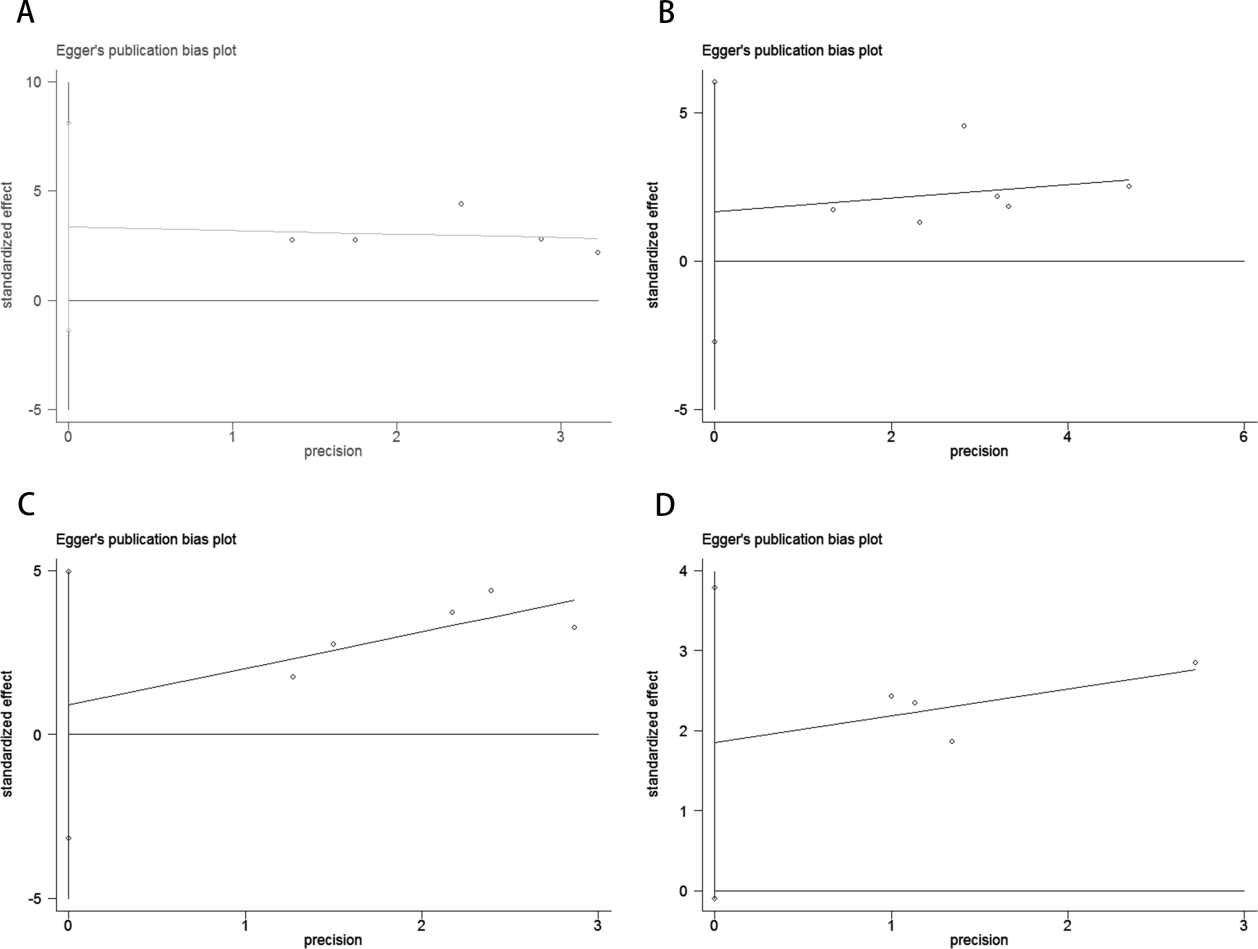
Publication bias test. **(A)** Egger tests for OS before ICI treatment, p = 0.110; **(B)** Egger tests for PFS before ICI treatment, p = 0.349; **(C)** Egger tests for OS after receiving ICI therapy.p = 0.531; **(D)** Egger tests for PFS after receiving ICI therapy. p = 0.055.

Publication bias was assessed using funnel plots, Egger’s linear regression, and Begg’s regression. Funnel plots for OS and PFS in MM patients receiving ICI therapy showed favorable symmetry (pre-treatment: OS, **Figure 3A**; PFS, **Figure 3B**; post-treatment: OS, **Figure 3C**; PFS, **Figure 3D**). The results of the Begg test indicated no significant publication bias for OS and PFS in MM patients before and after ICI treatment (pre-treatment: OS, p = 0.266, **Figure 4A**; PFS, p = 0.118, **Figure 4B**; post-treatment: OS, p = 0.266, **Figure 4C**; PFS, p = 0.118, **Figure 4D**). Similarly, the results from the Egger test suggested no significant publication bias for OS and PFS in MM patients before and after treatment (pre-treatment: OS, p = 0.266, **Figure 5A**; PFS, p = 0.118, **Figure 5B**; post-treatment: OS, p = 0.266, **Figure 5C**; PFS, p = 0.118, **Figure 5D**). These analyses indicate that the findings of this study are statistically significant and robust, without substantial interference from publication bias.

### 3.4 Sensitivity analysis

**Figure 6:**
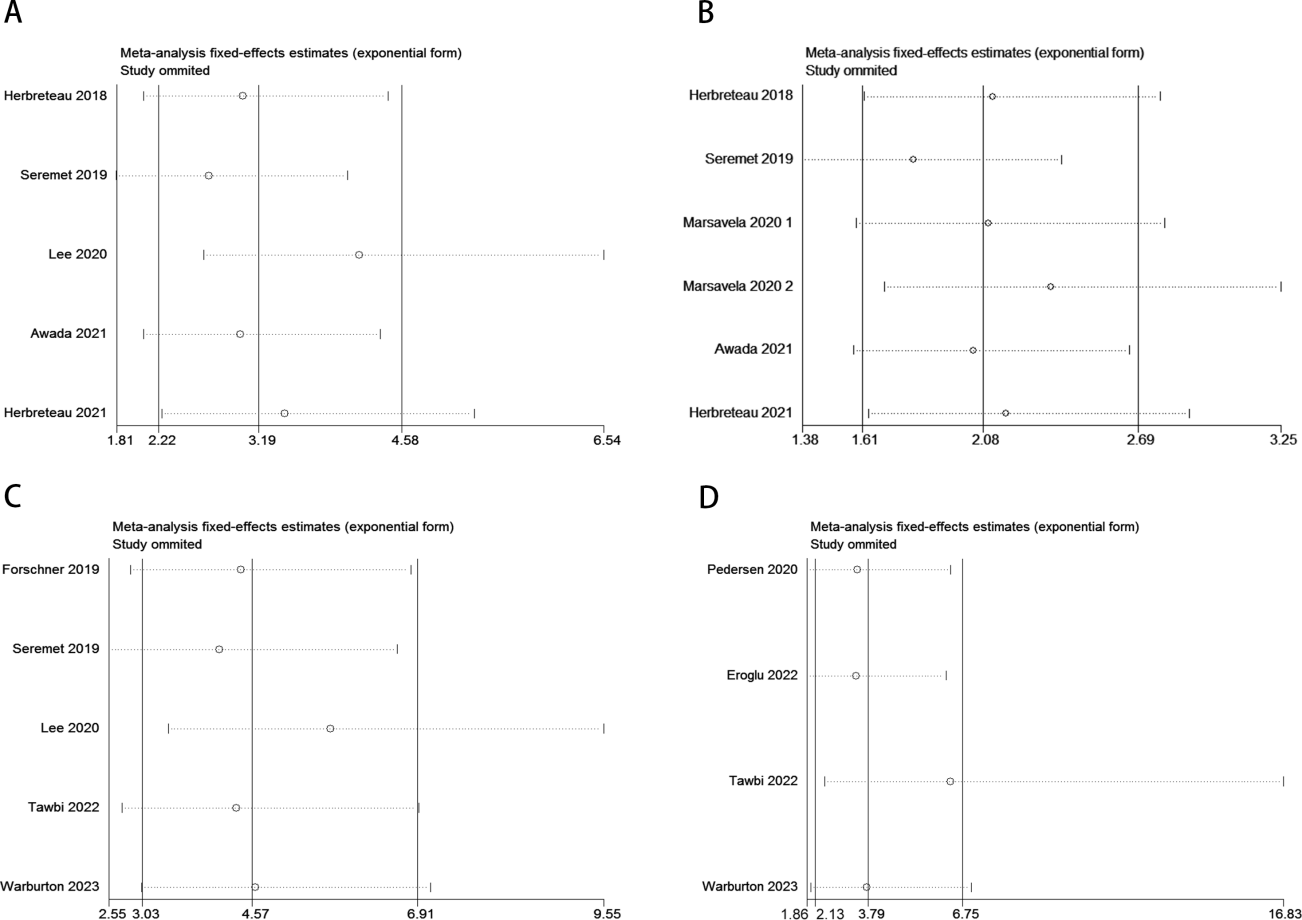
Sensitivity analysis for the pooled results between ctDNA and OS as well as PFS in MM patients before and after receiving ICI therapy. (Pre-treatment: OS: **A**; PFS: **B**; Post-treatment: OS: **C**; PFS: **D**).

Sensitivity analysis revealed that no individual study significantly influenced the observed effect size of the association between ctDNA and OS or PFS in MM patients before and after receiving ICI therapy. In this study, the removal of any single article did not result in significant changes, indicating the reliability of the results (**Figure 6**).

## 4 Discussion

Malignant melanoma is a highly invasive malignant skin tumor, commonly occurring in the skin, mucous membranes, and extremities. It is a major cause of skin cancer-related deaths, accounting for approximately 80% of such cases (37–39). In recent years, MM incidence has increased significantly, making it one of the fastest-growing malignancies worldwide (40). In the early stages, surgical excision is the primary treatment. However, once melanoma enters a rapid growth phase, prognosis deteriorates, with high mortality rates, and traditional radiotherapy and chemotherapy have limited effectiveness (41, 42). The advent of ICI has significantly improved treatment and prognosis for metastatic melanoma. Research shows that cytotoxic T-lymphocyte antigen-4 (CTLA-4) inhibitors, the first ICI approved by the U.S. Food and Drug Administration (FDA), can improve survival rates in patients with advanced melanoma (43, 44). However, the durable clinical responses induced by these therapies are observed in only a subset of patients. Therefore, to minimize adverse effects and further improve patient survival, biomarkers are urgently needed to guide clinical treatment decisions.

Circulating tumor DNA, DNA fragments shed into the bloodstream by apoptotic or necrotic tumor cells, has shown promise as a tumor-specific biomarker (45). It is non-invasive, easily accessible, suitable for repeated sampling, and can overcome the challenges of tumor heterogeneity, thus providing a valuable complement to tissue biopsies for clinical diagnosis and disease monitoring. Studies by the Pinzani team (46) revealed that ctDNA testing in MM patients has a sensitivity of 72% and a specificity of 89% compared to tumor tissue detection, with an 80% consistency with pathological results. Additionally, Haselmann et al. (47) found in a study of 187 MM patients that ctDNA assessment could be standardized before tumor recurrence, exhibiting higher specificity than serum S100 and lactate dehydrogenase, suggesting its diagnostic potential. Additionally, during immune checkpoint inhibitor (ICI) therapy, spatial CITE-seq can reveal the activation status of immune cells within the tumor tissue and their interactions with tumor cells (48). Meanwhile, ctDNA can reflect the overall tumor burden and treatment efficacy systemically. The combination of these two approaches offers a more accurate prediction of clinical outcomes in immune therapy. However, factors such as detection technology, sample origin, and disease staging can influence the accuracy of ctDNA testing, and its prognostic value in ICI-treated MM patients remains a subject of ongoing debate.

This study systematically reviewed the relationship between ctDNA and survival rates in ICIs-treated MM patients, analyzing 11 articles covering over 1,000 melanoma cases. Meta-analysis results showed a significant association between ctDNA fluctuations before and after ICIs treatment and the prognosis of MM patients. Compared with patients without detectable ctDNA, those with detectable or higher levels of ctDNA exhibited poorer OS and PFS. Despite the limited number of studies available, ctDNA testing shows potential in clinically assessing OS and PFS risk ratios, suggesting its value in monitoring immune therapy response (49, 50). Furthermore, no significant heterogeneity was observed in the included studies, as indicated by an acceptable I² statistic. Key factors affecting the results included study design, tumor staging, ctDNA measurement timing, and types of prognostic events considered. Although variability exists, the forest plot structure demonstrates stability in the pooled results, supporting ctDNA as an independent prognostic marker for advanced MM patients undergoing ICIs therapy. Another study suggests that ctDNA may help differentiate between true progression and pseudo-progression in MM patients receiving PD-1 antibody therapy. Early monitoring of ctDNA fluctuations could assist clinicians in identifying patients who are not responding to therapy, facilitating timely treatment adjustments and reducing unnecessary costs and ineffective treatment (51, 52). Atsuko et al. indicated that ctDNA could independently reflect the impact of adverse events on tumor burden during ICIs treatment (53). Although this study provides valuable insights into the relationship between ctDNA and prognosis in MM patients receiving ICIs therapy, several limitations must be considered. Firstly, the limited number of studies and relatively small sample sizes may restrict the generalizability of these findings. Furthermore, variability in study design, detection methods, and patient characteristics across studies could introduce bias, affecting the robustness of the results. Additionally, inconsistencies in the timing of ctDNA measurement and threshold definitions may impact the interpretation and comparability of results. Lastly, the short follow-up durations in some studies hinder a comprehensive assessment of the long-term prognostic value of ctDNA. Therefore, future research should focus on developing standardized ctDNA measurement protocols, including unified extraction and detection techniques, threshold definitions, and multicenter validation, to enhance predictive accuracy and reduce methodological variability. Moreover, integrating other omics data (such as genomics, proteomics, and immunomics) with long-term monitoring of ctDNA fluctuations may further improve the accuracy of predicting immune therapy responses. For example, combining spatial multi-omics sequencing technologies, such as DBiT ARP-seq and DBiT CTRP-seq, with multiplex immunofluorescence imaging techniques like CODEX to map immune responses could aid in monitoring disease progression (54). With these improvements, ctDNA has the potential to become a more reliable prognostic biomarker in clinical practice, supporting personalized immune therapy strategies.

## 5 Conclusions

This meta-analysis demonstrates a significant association between the presence of ctDNA and the prognosis of MM patients receiving ICI therapy, establishing ctDNA as a specific prognostic biomarker. Additionally, ctDNA shows potential as a tool for stratified diagnosis before treatment and as an effective indicator for monitoring treatment response and disease progression during therapy. Looking forward, we anticipate further high-quality research to provide strong evidence-based support for the clinical application of ctDNA in MM genetic testing.

## Data Availability

The original contributions presented in the study are included in the article/supplementary material. Further inquiries can be directed to the corresponding author.
